# Predicting aging trajectories of decline in brain volume, cortical thickness and fractional anisotropy in schizophrenia

**DOI:** 10.1038/s41537-022-00325-w

**Published:** 2023-01-03

**Authors:** Jun-Ding Zhu, Shih-Jen Tsai, Ching-Po Lin, Yi-Ju Lee, Albert C. Yang

**Affiliations:** 1 Institute of Brain Science, National Yang Ming Chiao Tung University, Taipei, Taiwan; 2 Department of Psychiatry, Taipei Veterans General Hospital, Taipei, Taiwan; 3 Institute of Neuroscience, National Yang Ming Chiao Tung University, Taipei, Taiwan; 4 Institute of Statistical Science, Academia Sinica, Taipei, Taiwan; 5 Digital Medicine and Smart Healthcare Research Center, National Yang Ming Chiao Tung University, Taipei, Taiwan; 6 Department of Medical Research, Taipei Veterans General Hospital, Taipei, Taiwan

**Keywords:** Biomarkers, Schizophrenia

## Abstract

Brain-age prediction is a novel approach to assessing deviated brain aging trajectories in different diseases. However, most studies have used an average brain age gap (BAG) of individuals with schizophrenia of different illness durations for comparison with healthy participants. Therefore, this study investigated whether declined brain structures as reflected by BAGs may be present in schizophrenia in terms of brain volume, cortical thickness, and fractional anisotropy across different illness durations. We used brain volume, cortical thickness, and fractional anisotropy as features to train three models from the training dataset. Three models were applied to predict brain ages in the hold-out test and schizophrenia datasets and calculate BAGs. We divided the schizophrenia dataset into multiple groups based on the illness duration using a sliding time window approach for ANCOVA analysis. The brain volume and cortical thickness models revealed that, in comparison with healthy controls, individuals with schizophrenia had larger BAGs across different illness durations, whereas the BAG in terms of fractional anisotropy did not differ from that of healthy controls after disease onset. Moreover, the BAG at the initial stage of schizophrenia was the largest in the cortical thickness model. In contrast, the BAG from approximately two decades after disease onset was the largest in the brain volume model. Our findings suggest that schizophrenia differentially affects the decline of different brain structures during the disease course. Moreover, different trends of decline in thickness and volume-based measures suggest a differential decline in dimensions of brain structure throughout the course of schizophrenia.

## Introduction

Schizophrenia is an essential health concern worldwide, with a global prevalence of ~1%. It affects the physical, mental, cognitive, and social domains^1,2^. Abnormalities in brain volume, cortical thickness, and fractional anisotropy (FA) are observed in individuals with schizophrenia, especially in the early stages^3–8^. However, whether these features of structural deterioration are present in patients with schizophrenia across different durations of illness remains unclear.

Estimation of brain age is an innovative approach that uses neuroimaging data and machine learning methods to predict aging trajectories in healthy participants^9^. The difference between brain age and chronological age, termed the brain age gap, can serve as a parameter for determining correlations with participants’ clinicodemographic characteristics^9^. T1-weighted MRI studies have used brain age to examine disease-related brain deterioration in schizophrenia^10–16^. Schnack and colleagues reported that the brain ages of individuals with schizophrenia were approximately 3.36 years higher than their chronological ages^10^. Another study reported that the average brain age (brain age gap = 2.56) was significantly higher in individuals with schizophrenia than in healthy controls and individuals with bipolar disorder^11^. One study used large-scale structural covariance networks and estimated that individuals with schizophrenia had higher brain age (brain age gap = 5.69 years) than that of healthy controls^13^. In addition, individuals with schizophrenia had higher brain age than their chronological age at early stage^12,15^. Notably, a recent study found a positive correlation between the brain age gap and cytokine TNFα in schizophrenia^16^. Constantinides et al. included 26 international cohorts via the ENIGMA consortium to test the brain age gap between individuals with schizophrenia and healthy controls based on 77 FreeSurfer features. They found that individuals with schizophrenia had a larger brain age gap than those of healthy controls with moderate effect size across multiple independent cohorts worldwide^17^.

A Few studies have used diffusion tensor imaging (DTI) to predict whether the brain aging trajectory in individuals with schizophrenia was aberrant compared with healthy individuals. Wang et al. reported individuals with schizophrenia had a significantly larger brain age gap than that of healthy controls after the age of 30^18^. Tønnesen and colleagues used the DTI features of the white matter structure and organization to predict brain age in individuals with schizophrenia and bipolar disorder^19^. They revealed that the brain age gaps in both groups of individuals were significantly larger than that in healthy controls^19^. In addition, they noted that the brain age model constructed using FA displayed higher sensitivity than that of models based on other features, such as axial diffusivity, mean diffusivity, and radial diffusivity^19^. A few studies have combined multimodal MRI to estimate brain age in individuals with schizophrenia^20,21^. The results demonstrated that the brain age of individuals with schizophrenia was higher than their chronological age^20,21^.

Taken together, the findings of both T1-weighted MRI and DTI studies have indicated that brain age is higher than chronological age in individuals with schizophrenia, suggesting that the neuroanatomical structure of individuals with schizophrenia has disease-related accelerated aging. However, most studies have attempted to predict brain age by using single-brain structural data derived from T1-weighted images or a single modality of brain imaging^10–12,17,19^. In addition, most studies have used an average brain age gap of individuals with schizophrenia of different durations for comparison with healthy participants^10,11,13,17,19,20^. Only a few studies have examined the brain age gap between patients with different stages of schizophrenia. Koutsouleris et al. found a linear increase in brain age gap from the at-risk, recent onset, and recurrent stages of individuals with schizophrenia^22^. Huang and colleagues indicated that the brain age gap of young individuals with schizophrenia was larger than that of healthy controls; however, the same result was not observed in middle-aged individuals with schizophrenia^21^. Demro et al. demonstrated that the disease course of psychotic illness was not significantly correlated with their brain age gap^14^. Chen et al. also found that the normalized predicted age difference did not show a significant correlation with the duration of illness in schizophrenia^23^. We found that previous studies had inconsistent results on the association between the brain age gap and duration of illness in individuals with schizophrenia^14,17,21,22^. The relevant studies are still limited. In addition, Kong et al. suggested that brain volume and cortical thickness did not share the overlapping regions completely in identifying morphometric changes^24^. Past studies have mentioned that different brain structural parameters (e.g., brain volume, cortical thickness, and FA) have different age-related aging trajectories across the lifespan^25–28^. Therefore, the goal of this study is to investigate the aging trajectories from different brain structural quantitative perspectives and further use these trajectory models to evaluate the impacts of disease courses on brain structures in individuals with schizophrenia. This method might help provide precise information about underlying disease mechanisms and clarify disease-related impacts on brain structures across different durations of illness in schizophrenia.

In this study, we (1) trained three brain-age prediction models based on brain volume, cortical thickness, and FA data from healthy controls and (2) investigated whether these models can be used to characterize the structural changes in schizophrenia at different durations of illness.

## Results

### Participants’ clinicodemographic characteristics

**Fig. 1:**
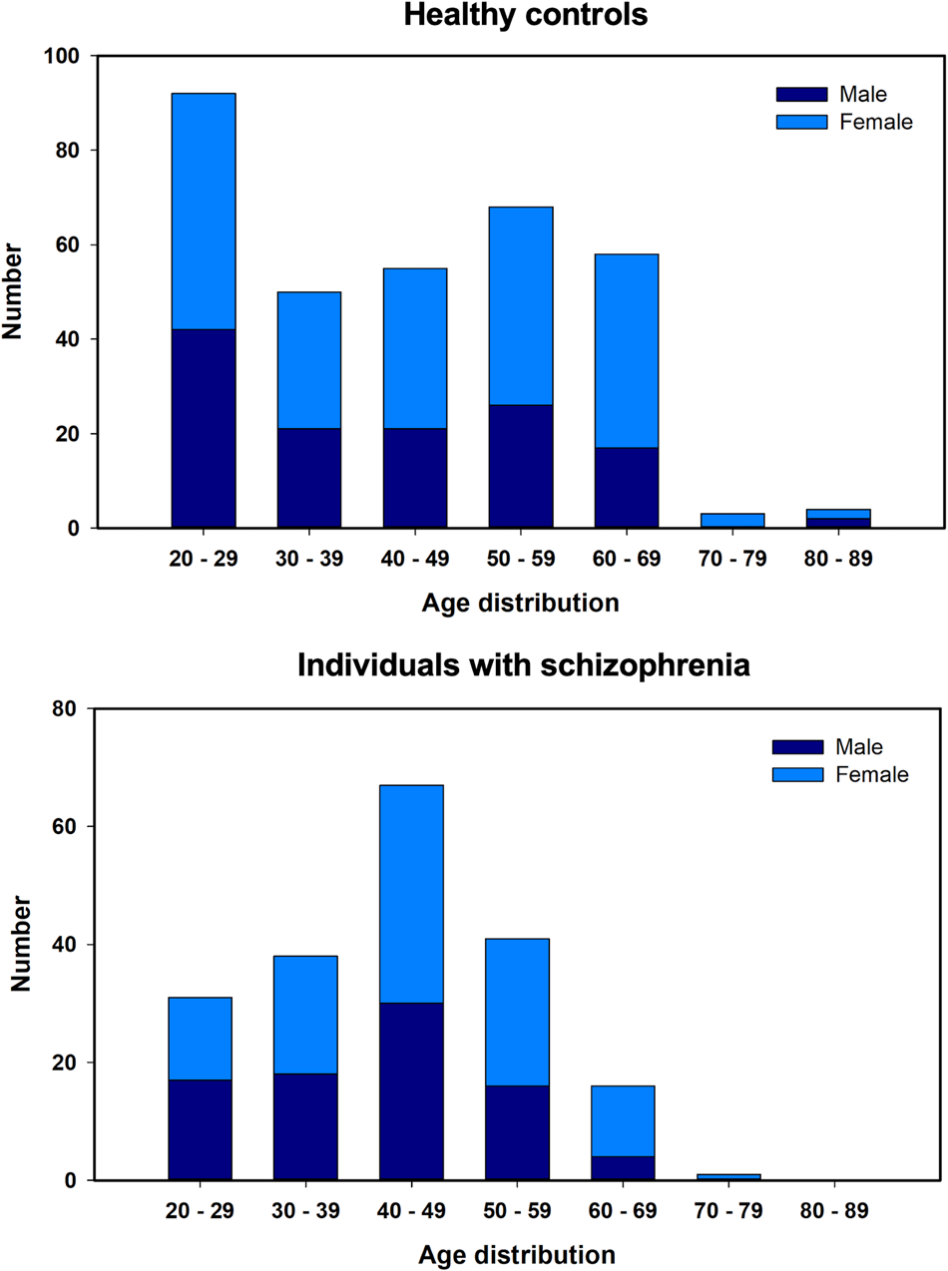
Age distributions of study participants. The upper panel presents the age distribution of healthy controls, and the lower panel illustrates the age distribution of individuals with schizophrenia.

We recruited 524 participants (194 with schizophrenia and 330 healthy controls). The two groups were balanced in terms of age (individuals with schizophrenia: 43.25 ± 11.93 years, range: 20–70; healthy controls: 43.49 ± 15.47 years, range: 20–84; *p* = 0.85) and sex (*p* = 0.28). Healthy controls had a higher average education level (*p* < 0.001) and MMSE score (*p* < 0.001) than those individuals with schizophrenia. The average duration of illness in individuals with schizophrenia was 15.56 ± 10.30 years (range: 0–38 years; Table 1). Table 1 and Fig. 1 also present the age distributions of the two groups.

**Table 1:** Clinicodemographic characteristics of individuals with schizophrenia and healthy controls.

Characteristics	SZ (*n* = 194)	HC (*n* = 330)	Statistic (t or *χ*^2^)	*p* value
Sex			1.13	0.28 ^b^
Male, *n* (%)	85 (43.8%)	129 (39.1%)		
Female, *n* (%)	109 (56.2%)	210 (60.9%)		
Age, year	43.25 ± 11.93	43.49 ± 15.47	0.20	0.85 ^a^
(Range)	(20–70)	(20–84)		
20–29			0.78	0.38 ^b^
Male/female, *n*	17/14	42/50		
Total, *n* (%)	31 (16.0%)	92 (27.9%)		
30–39			0.25	0.62 ^b^
Male/ female, *n*	18/20	21/29		
Total, *n* (%)	38 (19.6%)	50 (15.2%)		
40–49			0.54	0.46 ^b^
Male/ female, *n*	30/37	21/34		
Total, *n* (%)	67 (34.5%)	55 (16.6%)		
50–59			0.01	0.94 ^b^
Male/ female, n	16/25	26/42		
Total, *n* (%)	41 (21.1%)	68 (20.6%)		
60–69			0.12	0.98 ^b^
Male/ female, *n*	4/12	17/41		
Total, *n* (%)	16 (8.3%)	58 (17.6%)		
70–79			NA	NA
Male/female, *n*	0/1	0/3		
Total, *n* (%)	1 (0.5%)	3 (0.9%)		
80–89			NA	NA
Male/female, *n*	0/0	2/2		
Total, *n* (%)	0 (0.0%)	4 (1.2%)		
Education level, year	12.50 ± 3.55	15.85 ± 3.85	9.90	<0.001 ^a^
MMSE	26.81 ± 3.39	28.96 ± 1.01	8.59	<0.001 ^a^
Duration of illness, year	15.56 ± 10.30	NA	NA	NA
<1				
Male/female, *n*	5/5	NA	NA	NA
Total, *n* (%)	10 (5.2%)			
1–5				
Male/female, *n*	13/15	NA	NA	NA
Total, *n* (%)	28 (14.4%)			
6–10				
Male/female, *n*	25/19	NA	NA	NA
Total, *n* (%)	44 (22.7%)			
11–20				
Male/female, *n*	18/28	NA	NA	NA
Total, *n* (%)	46 (23.7%)			
>20				
Male/female, *n*	24/42	NA	NA	NA
Total, *n* (%)	66 (34.0%)			
PANSS score				
Total	42.04 ± 10.76	NA	NA	NA
Positive symptoms	10.74 ± 3.37	NA	NA	NA
Negative symptoms	10.04 ± 3.76	NA	NA	NA
General psychopathology symptoms	21.26 ± 5.18	NA	NA	NA
CPZ equivalent dosage^c^	402.42 ± 324.27	NA	NA	NA

Data were mean ± SD or *n* (%) unless specified otherwise.

*SZ* patients with schizophrenia, *HC* healthy controls, *MMSE* mini-mental state examination, *PANSS* positive and negative syndrome scale, *CPZ* chlorpromazine.

^a^Independent *t*-test, significance level = 0.05.

^b^Chi-square test, significance level = 0.05.

^c^Only 161 patients with schizophrenia had verified medication records.

### Brain-age prediction

We calculated the mean absolute error (MAE) and mean Pearson’s correlation between brain age and chronological age after repeating the whole cross-validation procedure one thousand times in the training dataset to assess the model performances. The moderate to high correlations between brain age and chronological age in the training dataset in all three brain-age prediction models before brain age correction were observed: brain volume (mean MAE: 6.86 ± 0.13, 95% confidence intervals: 6.85–6.87; mean r: 0.832 ± 0.007, 95% confidence intervals: 0.832–0.833), cortical thickness model (mean MAE: 9.12 ± 0.15, 95% confidence intervals: 9.11–9.13; mean r: 0.688 ± 0.012, 95% confidence intervals: 0.687–0.689), and FA model (mean MAE: 8.54 ± 0.16, 95% confidence intervals: 8.53–8.55; mean r: 0.749 ± 0.009, 95% confidence intervals: 0.748–0.749).

**Fig. 2:**
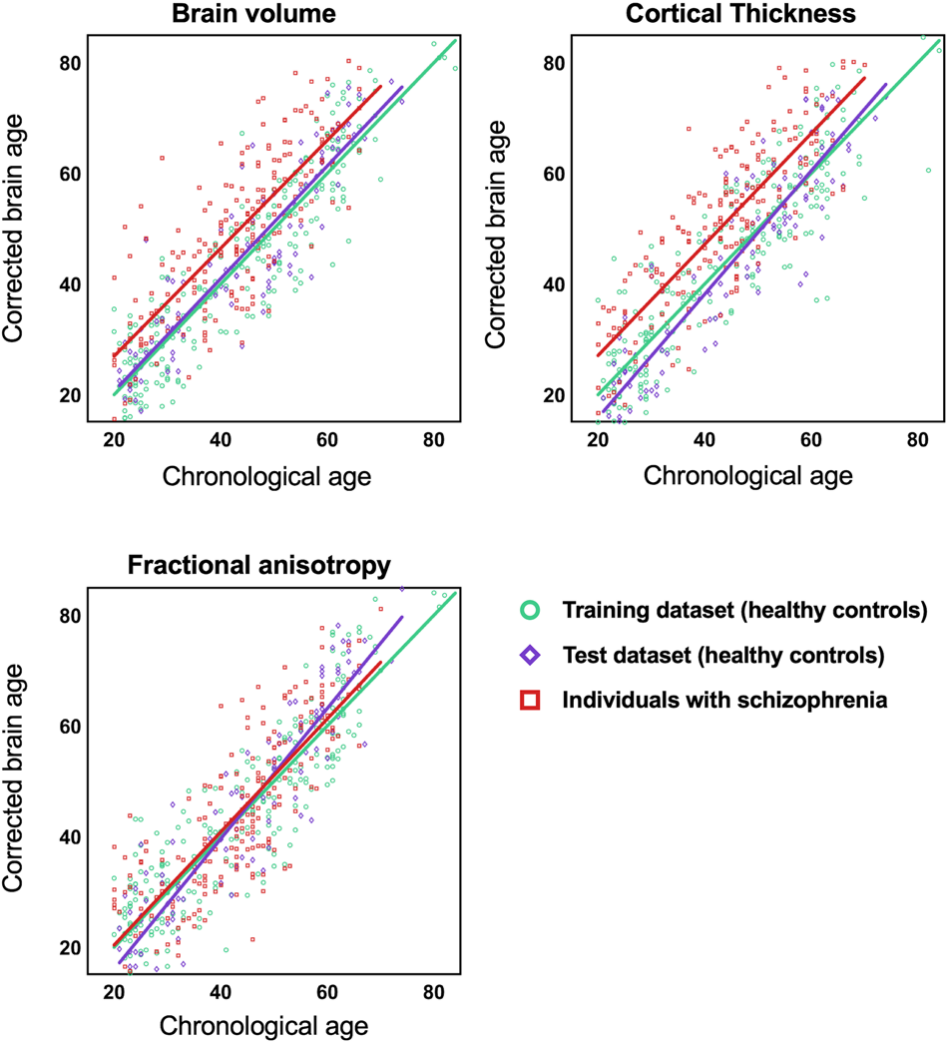
Performances of three brain-age prediction models in healthy controls (training and hold-out test datasets) and individuals with schizophrenia. The scatter plots present chronological age and corrected brain age in three different brain features. The green line represents the best linear fit in the training dataset. The purple line represents the best linear fit in the hold-out test dataset. The red line represents the best linear fit in individuals with schizophrenia.

Next, we applied the trained models to predict brain ages of hold-out test and schizophrenia datasets and calculated the MAE and Pearson’s correlation between corrected brain age and chronological age after brain age correction in three datasets. Finally, we obtained the following results: brain volume (training dataset: MAE = 5.60, *r* = 0.91; hold-out test dataset: MAE = 5.46, *r* = 0.91; schizophrenia dataset: MAE = 8.73, *r* = 0.80), cortical thickness (training dataset: MAE = 6.16, *r* = 0.90; hold-out test dataset: MAE = 6.67, *r* = 0.91; schizophrenia dataset: MAE = 9.06, *r* = 0.83), and FA (training dataset: MAE = 6.18, *r* = 0.90; hold-out test dataset: MAE = 6.60, *r* = 0.92; schizophrenia dataset: MAE = 7.70, *r* = 0.81) (Fig. 2).

### Group differences in brain age gap across different durations of illness

We compared the differences in the brain age gap between each group of schizophrenia and age- and sex-matched healthy controls (independent hold-out test dataset) across different brain structures (see Supplementary Table 1 for more details).

**Fig. 3:**
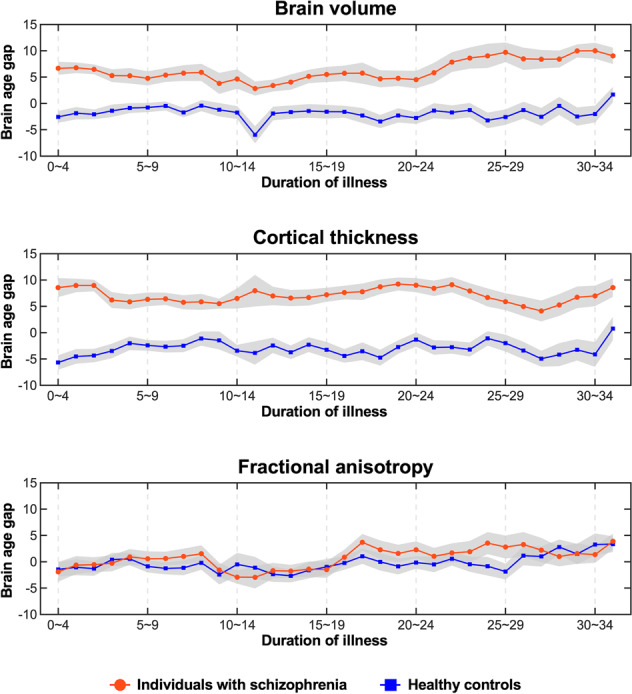
The brain age gaps of the individuals with schizophrenia and healthy controls across different durations of illness in three models. The colored lines represent the means of the brain age gap across the duration of illness in two groups, and the shaded areas represent the standard error of the mean.

**Fig. 4:**
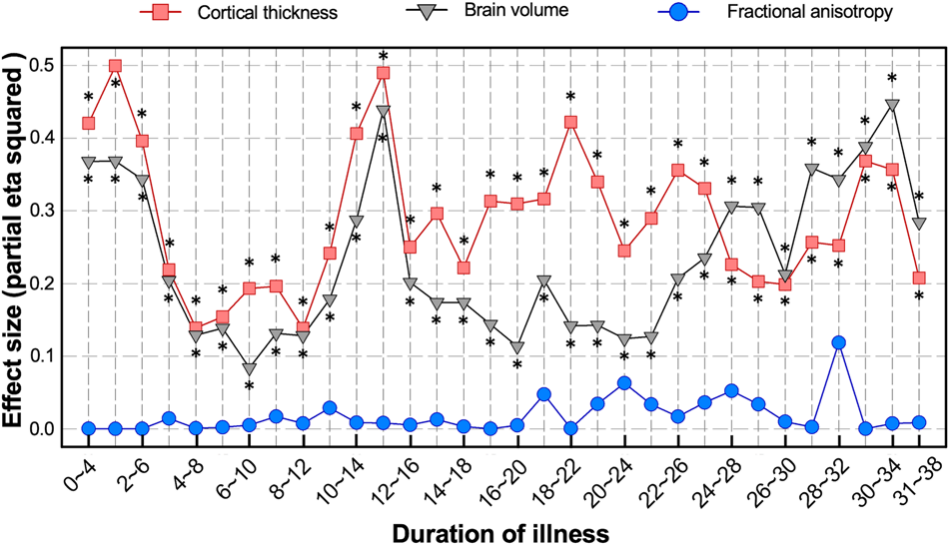
Effect sizes (partial eta squared) from comparisons of the brain age gap across different durations of illness between individuals with schizophrenia and healthy controls (hold-out test dataset). * significant difference after Bonferroni correction.

The brain volume model revealed that individuals with schizophrenia had a larger brain age gap than healthy controls across different durations of illness (*p* < 0.01), with medium to large effect sizes (partial eta squared range: 0.08–0.45; Supplementary Table 2). In addition, from ~22 years after schizophrenia onset, the effect sizes tended to increase progressively, suggesting that a longer duration of illness was associated with a more deviated brain aging trajectory in the brain volume model (Figs. 3, 4).

In the cortical thickness model, the individuals with schizophrenia had a significantly larger brain age gap than that of healthy controls across different durations of illness after Bonferroni correction (*p* < 0.01) with large effect sizes (partial eta squared range: 0.14–0.50). These findings were similar to those of the brain volume model (Supplementary Table 3). However, in comparison to that of the brain volume model, the deviation of the brain aging trajectory from the initial stage of the disease was more intense in the cortical thickness model until 24 years after disease onset (Figs. 3, 4).

In the FA model, no significant differences were found between healthy controls and individuals with schizophrenia across different durations of illness (Supplementary Table 4). These findings suggest that the FA of individuals with schizophrenia did not differ from that of healthy controls across different durations of illness (Figs. 3, 4).

### Association of brain age gaps with clinical characteristics in schizophrenia

For the severity of symptoms, no significant correlation was found between the brain age gap and the Positive and Negative Syndrome Scale (PANSS) total score in the three brain-age prediction models (brain volume: *p* = 0.70; cortical thickness: *p* = 0.97; FA: *p* = 0.41). In addition, PANSS subscales (i.e., positive, negative, and general psychopathology symptoms) were also not correlated to the brain age gaps in the three models (positive symptoms: brain volume: *p* = 0.39; cortical thickness: *p* = 0.80; FA: *p* = 0.87. negative symptoms: brain volume: *p* = 0.45; cortical thickness: *p* = 0.69; FA: *p* = 0.21. general psychopathology symptoms: brain volume: *p* = 0.42; cortical thickness: *p* = 0.85; FA: *p* = 0.36).

For the chlorpromazine (CPZ) equivalent dosages, only participants with verified medication records (*N* = 161) were included in the analysis. The results indicated that there were no significant correlations between CPZ equivalent dosages and brain age gaps in the three models (brain volume: *p* = 0.84; cortical thickness: *p* = 0.20; FA: *p* = 0.90).

## Discussion

The present study has three main findings. First, our three brain-age prediction models allowed reliable predictions of chronological age in healthy controls. Second, we identified larger brain age gaps across different durations of illness in the brain volume and cortical thickness models in schizophrenia. In contrast, the FA model indicated there was no significant deviated brain aging trajectory between individuals with schizophrenia and healthy controls across different durations of illness. Third, the deviation of the brain aging trajectory at the early stage of individuals with schizophrenia was strongest in the cortical thickness model. However, the deviation of the brain aging trajectory from two decades (~24 years) after schizophrenia onset was most pronounced in the brain volume model.

The three models were trained in this study using the brain volume, cortical thickness, and FA value from 230 healthy controls by the Gaussian process regression method with fivefold cross-validation. After repeating the whole cross-validation procedure one thousand times, the estimated brain ages of the training dataset had moderate to high correlations with the chronological ages, with mean MAEs ranging from 6.68 to 9.12 years in the three models. Also, the estimated brain ages of the hold-out test dataset were highly correlated with the chronological ages, with MAEs ranging from 5.46 to 6.67 years after brain age correction in the three models. The results were consistent with those of brain-age estimation studies^19,29^. Tønnesen and colleagues extracted all features of DTI (i.e., FA, axial diffusivity, mean diffusivity, and radial diffusivity) from healthy controls to train the models. The main model, including all features, had a high correlation between brain age and chronological age, and the MAE was 6.49 years^19^. Another study extracted T1-weighted, T2-weighted, and arterial spin labeling data to estimate brain age in healthy controls. The brain ages had moderate to high correlations with chronological ages (correlation coefficients: 0.56–0.88), and the MAEs ranged from 6.4 to 11.2 years in all trained models^29^. Gaussian process models are beneficial for clinical practice and multiple neuroimaging applications, including brain age prediction, cortical maps, white matter fiber clustering, disease status, and clinical outcomes through classification and regression^30–36^. Therefore, the method used in this study to train the brain-age prediction model is reliable. We also found that the hold-out test dataset outperformed the training dataset in the brain volume model after brain age correction. Similar results could be found in previous brain age studies^13,34,37^. In this study, we randomly divided 330 healthy controls into the training and test datasets in a ratio of 7 to 3. There were no statistically significant differences in the proportion of males and females between the two datasets in each age group in this study. However, the proportion of total participants in each age group distribution was different in the training and hold-out test datasets (Supplementary Table 5 and Supplementary Fig. 1), which might lead to a lower MAE in the hold-out test dataset than that in the training dataset in the brain volume model.

We found significantly deviated brain aging trajectories in brain volume and cortical thickness across different durations of illness in individuals with schizophrenia as compared with those of healthy controls. Koutsouleris et al. found that the brain age gap of brain volume in individuals with schizophrenia progressively increased after disease onset^22^. One T1-weighted MRI study used the brain age method to estimate the brain age gap in the early stage of schizophrenia (average duration of illness = 4.61 years) and demonstrated that individuals with schizophrenia had larger brain age gaps than those of healthy controls^12^. A review study revealed a decline in multiple gray matter regions on structural MRI in both patients with first-episode psychosis and those with chronic schizophrenia^4^. The longitudinal studies demonstrated that brain structures in patients with schizophrenia deteriorated in the early stage after disease onset^10,38^. Cropley et al. found that from the age of 23, individuals with schizophrenia showed a significant reduction in total brain volume, reaching a peak of 8% loss relative to healthy controls at the age of 70^3^. These findings agree with our results. Our data indicated that brain aging, in terms of brain volume and cortical thickness deterioration, occurs continuously as the disease progresses and is reflected as an increasing brain age gap.

In the FA model, no significant differences were observed in the brain age gap between individuals with schizophrenia and healthy controls across different durations of illness. One study extracted the FA value to estimate brain age in schizophrenia and revealed that individuals with schizophrenia (corrected brain age gap: 0.24 ± 9.92) had a higher brain age gap than that of healthy controls (corrected brain age gap: –3.23 ± 8.66)^19^. Our results show that the corrected brain age gaps between individuals with schizophrenia and healthy controls (hold-out test dataset) were 0.91 ± 8.76 years and 0.37 ± 7.91 years, respectively. The estimated brain age of healthy controls in the previous study was younger than the chronological age. However, the estimated brain age of our healthy controls was close to their chronological age, which might be a possible reason for the inconsistent results. Cropley et al. reported that individuals with schizophrenia significantly declined in global FA from age 35 and progressively worsened until the oldest age^3^. A DTI study showed that brain age was significantly higher in patients with schizophrenia after the age of 30. In contrast, accelerated brain aging was not observed in patients with schizophrenia before the age of 30^18^. These results are similar to our findings. In the FA model, individuals with schizophrenia showed a progressive trend toward deviation of aging trajectory of FA around 17 years after disease onset (mean age = 45.78), although there was no significant difference in the statistical analysis. Previous DTI studies have demonstrated that individuals with chronic schizophrenia had a significant reduction in FA values compared with healthy controls in the whole brain and in the genu of the corpus callosum, right forceps minor, and left inferior longitudinal fasciculus; however, similar results were not observed in individuals with first-episode schizophrenia^39–41^. These results may suggest that FA abnormalities might occur in middle age or in chronic patients with schizophrenia.

The degrees of deviated brain aging trajectory in the brain volume and cortical thickness models varied at different stages of schizophrenia. The deviation at the initial stage of the disease was strongest in the cortical thickness model, but the deviation from approximately 24 years after disease onset was most vigorous in the brain volume model. A study demonstrated that cortical thickness, cortical surface area, gray/white matter intensity contrast, and curvature might jointly drive changes in brain volume, leading to potential differences between brain volume and cortical thickness^24^. Previous studies have shown that cortical thickness deteriorated more than the surface area in the initial stages of individuals with schizophrenia, and this factor might be the main reason for the reduction of the whole brain volume in schizophrenia during the initial phase^42,43^. However, the reduction of brain volume in chronic individuals with schizophrenia might be driven jointly by different brain structural parameters (e.g., cortical thickness, surface area, gray/white matter intensity contrast, and curvature) rather than being caused primarily by cortical thinning^24,44^. Therefore, we speculated that brain volume was affected by different brain structural parameters, such as cortical thickness, surface area, gray/white matter intensity contrast, and curvature. Taken together, our findings of differentially deviated brain aging trajectories in brain volume before and after two decades of disease course (~24 years) might have different causes, namely, cortical thinning in the earlier stage and joint deterioration in different brain structural parameters (e.g., cortical thickness, surface area, gray/white matter intensity contrast, and curvature) in the chronic stage. These causes might explain the larger brain age gap with larger effect sizes in the cortical thickness model than in the brain volume model in the early stage of schizophrenia. In the chronic stage, the brain age gap in the brain volume model began to be larger than that in the cortical thickness model, and the effect sizes in the brain volume model became more prominent as the duration of the illness advanced. Individuals with schizophrenia experience an abnormal neuronal pruning process, including the reduction of synaptic products or pyramidal layer thickness, which might lead to an accelerated process of cortical thinning^45–47^. However, the nature of biological changes that may cause an alteration in cortical surface area or brain tissue intensity contrast in the pathological process of individuals with schizophrenia remains unclear^44,48^. In this study, it is challenging to evaluate molecular and cellular alterations of individuals with schizophrenia with magnetic resonance imaging methodology. More relevant studies are needed in the future to validate our findings.

Finally, our results showed that there were no significant correlations between the brain age gaps with the severity of symptoms and CPZ equivalent dosages in three trained models. A recent study found that a larger brain age gap in schizophrenia could reflect cognitive decline rather than the severity of clinical symptoms^15^. Walton et al. reported that positive symptoms had an inverse relationship with cortical thickness in the superior temporal gyrus, and negative symptoms had a negative correlation with cortical thickness in the prefrontal cortex after controlling for age, sex, and site in their meta-analysis studies^49,50^. We used the whole-brain data as features to train three brain-age prediction models. This might reduce the sensitivity of brain age gaps to the severity of symptoms. In addition, most individuals with schizophrenia in our study were chronic and symptom-stable patients. These factors might lead to no significant correlations between brain age gaps and the severity of symptoms in the three models. A previous meta-analysis study found that high antipsychotic dosage had a negative relationship with parietal lobe volume and a positive relationship with basal ganglia volume^51^. According to previous results, antipsychotic medication use might be only related to impacts on specific brain regions^51^. However, in the present study, we did not focus on specific brain regions to explore the alterations in brain structures caused by antipsychotic medication use in individuals with schizophrenia. A recent study^52^ showed that early antipsychotic medication use might reduce the brain age gap in patients with first-episode schizophrenia. In contrast, the illness duration of the participants was longer in our study (mean: 15.56 years, ranging from 0 to 38 years), and our result showed a lack of association between medication and the brain age gap, which is consistent with the previous studies^17,18,23^. Therefore, we speculate that the brain age gap may be more sensitive to first-episode schizophrenia patients with early medication use but less sensitive to chronic schizophrenia patients with antipsychotic medication use.

The strengths of this study are that we trained three brain-age prediction models on three brain structural features. In addition, we used a 5-year sliding window to divide individuals with schizophrenia into multiple groups to explore the differences in the brain age gap between healthy controls and individuals with schizophrenia across different durations of illness. The previous studies^53–58^ have used the sliding time window method in different diseases (e.g., neurological^54^, psychiatric disorders^55,56^, and SARS-CoV-2^57,58^). This method could further explore the time-specific (e.g., age or duration of illness) changes in the brain of participants. With the sliding time window approach, we could construct similar aging trajectories in cross-sectional studies as in longitudinal studies, even if we lack longitudinal data. This approach also helped us clarify the differential impacts of schizophrenia on brain volume, cortical thickness, and FA across different durations of illness.

However, the present study still had some limitations. First, the present study used a cross-sectional design to explore the disease-related aging trajectories and alterations in the brain structures in schizophrenia. To have a clear understanding of the disease-related alteration in the brain structures, longitudinal prospective studies including individuals at ultra-high risk of psychosis and individuals with first-episode schizophrenia are required. Second, the individuals with schizophrenia ranged in age from 20 to 70 years. We could not investigate the disease-related impacts on gray or white matter of schizophrenia in early adolescence. Future studies including younger participants could provide a more comprehensive understanding of the pathological process of schizophrenia. Third, MMSE was performed to assess the overall cognitive function of individuals with schizophrenia in this study. However, this tool might be less sensitive to overall cognitive ability because of ceiling effects. Future studies to include more cognitive assessments may help understand the comprehensive cognitive ability of individuals with schizophrenia. Finally, we could not completely rule out the effects of antipsychotic drugs on the brain structures of individuals with schizophrenia. Further research should recruit drug-naïve participants to examine the impacts of antipsychotic medication on brain structures in individuals with schizophrenia.

In conclusion, this study comprehensively examined the deviation of the brain age gaps in terms of brain volume, cortical thickness, and FA in individuals with schizophrenia across different durations of illness by using a machine learning approach. In addition, our results suggested that the brain age gap can reflect the deterioration of brain structures. Therefore, using a machine learning approach to predict brain age is a promising method for detecting altered aging trajectories in neuropsychiatric disorders. We observed that both brain volume and cortical thickness had accelerated deterioration right from disease onset, whereas the brain age gap in terms of FA did not differ from that of healthy controls across different durations of illness. The deviation of the brain aging trajectory at the initial stage of the disease was strongest in the cortical thickness model; however, the deviation of the brain aging trajectory from two decades after disease onset (~24 years) was most vigorous in the brain volume model. Together, these findings suggest that schizophrenia has different impacts on the decline of different brain structures. Notably, the different trends of decline in thickness and volume-based measures suggest a differential decline in dimensions of brain structure throughout the course of schizophrenia. Future studies should extract different brain features from multiple neuroimaging modalities to provide more comprehensive insights into the neuropathology of schizophrenia.

## Methods

### Participants

We recruited 194 individuals with schizophrenia and 330 healthy controls. Each individual with schizophrenia was diagnosed according to the Diagnostic and Statistical Manual of Mental Disorders, Fourth Edition, by two psychiatrists. The cognitive function of all participants was assessed with the Mini-Mental State Examination (MMSE)^59^. In addition, the severity of symptoms was further assessed in individuals with schizophrenia with the PANSS^60^. Individuals with schizophrenia who had a history of other psychiatric disorders or neurological diseases were excluded. The psychiatrists retrieved the medication information of the individuals with schizophrenia from their medical records. This study was approved by the Institutional Review Board of National Yang Ming Chiao Tung University, and all participants provided written informed consent.

### Image acquisition

All participants were scanned with a 3 T MRI scanner (Siemens Magnetom Tim Trio, Erlangen, Germany). The DTI images were acquired with a single-shot spin-echo EPI sequence in the axial plane, with the following parameter settings: repetition time (TR) = 11,000 ms; echo time (TE) = 104 ms; the number of excitations = 3; matrix size = 128 × 128; field of view = 26 cm; slices = 70; slice thickness = 2.0 mm; and b-value = 1000 s/mm^2^. Thirty isotropic diffusion directions and three non diffusion weighted T2 images were obtained per participant. For T1-weighted MR scanning, the images were obtained using a sagittal 3D magnetization-prepared rapid gradient echo (MPRAGE) sequence, with the following parameter settings: TE = 3.5 ms; matrix size = 256 × 256; slices = 192; slice thickness = 1 mm; and voxel size = 1.0 × 1.0 × 1.0 mm^3^. The DTI and T1-weighted MRI scanning protocols were consistent with our previous study^61^.

### Image processing

**Fig. 5:**
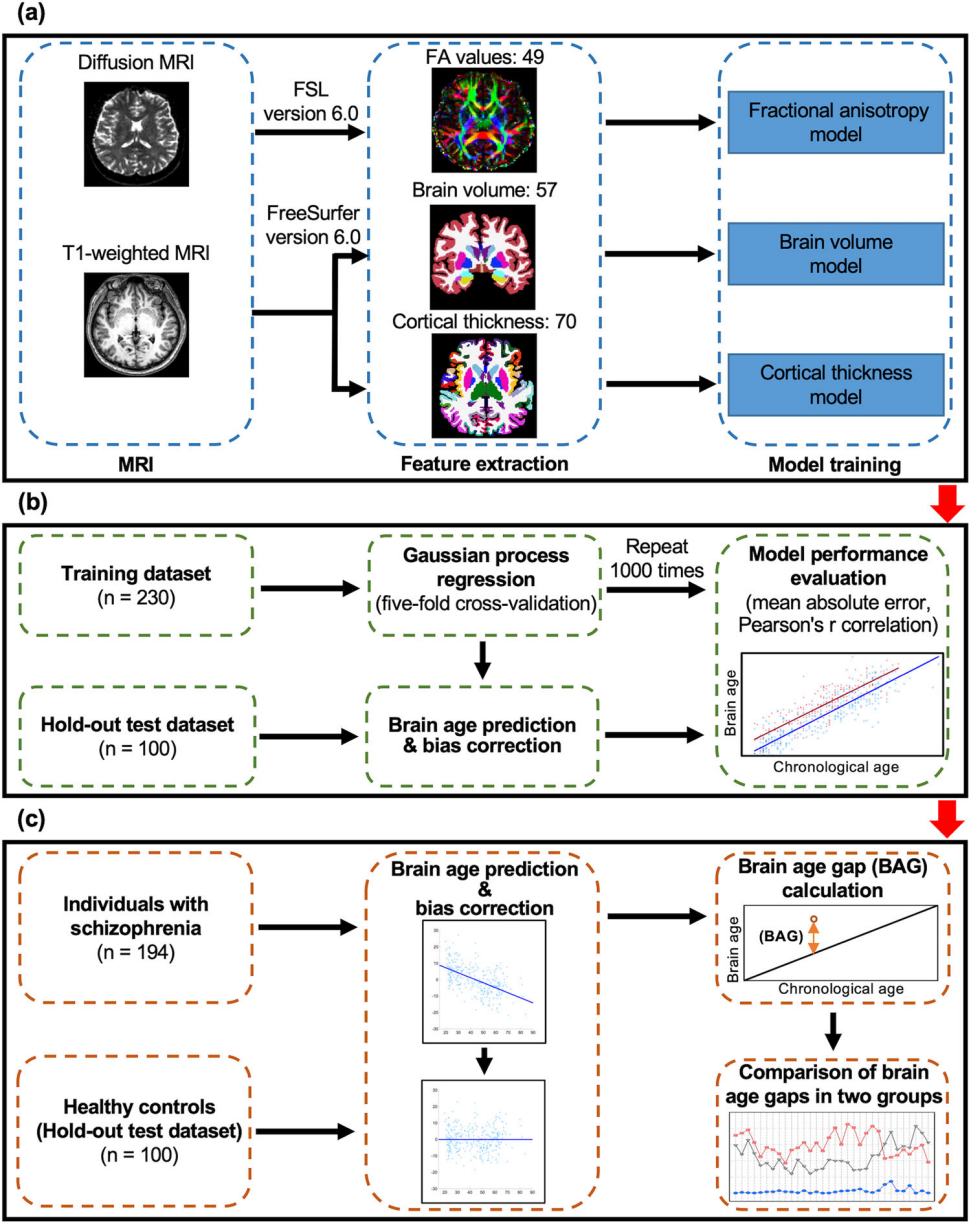
Illustration of the three trained brain-age prediction models. **a** The preprocessing and analysis of brain structural data. DTI data were preprocessed with the FSL toolbox, which was also used to calculate the 49 FA values. T1-weighted images were used to extract the 57 values of brain volume and the 70 values of cortical thickness with the FreeSurfer software suite. **b** The Gaussian process regression approach with fivefold cross-validation was used to train the models on the training dataset of 230 healthy controls. The models were assessed with MAE and with the correlation between the corrected brain age and chronological age after repeating the whole cross-validation procedure one thousand times in the training dataset. The hold-out test dataset of 100 healthy controls was utilized to test three trained models. **c** The brain ages of individuals with schizophrenia were estimated with the three models with bias correction. We performed the ANCOVA to compare the brain age gaps between the two groups across different durations of illness based on three different trained models. FA fractional anisotropy, MAE mean absolute error, BAG brain age gap.

Brain volume and cortical thickness were calculated using FreeSurfer’s standard auto-reconstruction algorithm^62–64^ (version 6.0.0, http://surfer.nmr.mgh.harvard.edu/). First, T1-weighted images were preprocessed to eliminate all nonbrain tissue, transformed into Talairach space, and segmented into gray matter and white matter. Next, the cortical surface model was reconstructed to locate the gray/white matter and gray/CSF boundaries. The values of cortical thickness were obtained by calculating the shortest distance between the two boundaries. A trained research assistant carefully inspected each image and manually corrected segmentation errors. A total of 70 values of cortical thickness in bilateral hemispheres were computed using the Desikan–Killiany atlas. We also acquired 57 values of brain volume by FreeSurfer’s automated segmentation (i.e., aseg) (Fig. 5a).

The FMRIB Software Library v6.0 (FSL)^65^ was used to preprocess the raw DTI data. The eddy tool was used to correct eddy currents and movements in the DTI data^66^. Brain tissue was extracted, and nonbrain tissue was removed with the brain extraction tool^67^. Next, the eddy-corrected data were fit into a tensor model at each voxel to create FA images. In the tract-based spatial statistics pipeline, all FA images were registered and aligned using the FMRIB58_FA standard-space image as the target. These images were normalized into an MNI152 standard space^68^. From these normalized images, FA values were calculated with the JHU-ICBM-labels-1mm atlas to obtain 48 FA values as regions of interest. We also averaged 48 FA values to acquire a mean FA value for each participant, which yielded 49 FA values as features in this study (Fig. 5a).

### Brain-age prediction

The healthy controls were randomly divided into a training dataset (*n* = 230) and an independent hold-out test dataset (*n* = 100) at a ratio of 7 to 3 (see Supplementary Table 5 for more details). We used the Regression Learner Tool in MATLAB R2021a (MathWorks, Natick, MA, USA) to perform the machine learning analysis. We trained three models based on the brain volume (57 features), cortical thickness (70 features), and FA (49 features) of the training dataset by using Gaussian process regression^30,31,34^ with fivefold cross-validation. Gaussian process regression is a nonparametric regression model, and this algorithm is based on the Bayesian approach^69^. The combination of T1-weighted MRI data and the Gaussian process regression method showed good performance in brain age prediction^31^. The Gaussian process regression algorithm was performed to train brain-age prediction models with an exponential kernel function, and we used the default values as model hyperparameters (basis function: constant; use isotropic kernel: true; kernel scale: automatic; signal standard deviation: automatic; sigma: automatic; standardize: true; optimize numeric parameters: true). Subsequently, the hold-out test dataset was utilized to test three trained models, which were also applied to the schizophrenia dataset to obtain an estimate of their brain age. A recent study found bias in brain age estimation in different age groups: brain ages tend to be overestimated in younger adults, while they tend to be underestimated in older adults^70^. To eliminate this bias, we corrected the estimated brain age according to the studies’ recommendations^70,71^. First, we fitted the brain age and chronological age using

1 $${{{{{\mathbf{Brain}}}}}}\,{{{{{\mathbf{age}}}}}} = \alpha \times \Omega + \beta$$

The coefficient Ω represents the chronological age, *α* represents the slope, and *β* represents the intercept. Second, we applied the derived values of *α* and *β* to correct the brain age using the following formula:

2 $${{{{{\mathbf{Corrected}}}}}}\,{{{{{\mathbf{brain}}}}}}\,{{{{{\mathbf{age}}}}}} = {{{{{\mathbf{Brain}}}}}}\,{{{{{\mathbf{age}}}}}} + \left[ {\Omega - \left( {\alpha \times \Omega + \beta } \right)} \right]$$

The three trained models were assessed with the mean absolute error (MAE) and mean Pearson’s correlation between brain age and chronological age after repeating the whole cross-validation procedure one thousand times in the training dataset (Fig. 5b). We also calculated the MAE and Pearson’s correlation between corrected brain age and chronological age after brain age bias correction in three datasets.

Next, we calculated the brain age gap by subtracting the chronological age from the corrected brain age for each participant of the hold-out test dataset and the schizophrenia dataset (Fig. 5c).

### Group comparison of the brain age gap across different durations of illness

The duration of illness of individuals with schizophrenia in this study ranged from 0 to 38 years. To investigate the acceleration of brain aging in schizophrenia in terms of brain volume, cortical thickness, and FA across the different durations of illness, we divided the individuals with schizophrenia into multiple groups using a 5-year sliding time window for the duration of illness with a 1-year interval step. For instance, individuals with a duration of illness from <1 year to <5 years were defined as the group of 0–4 years, and those with a duration of illness from <2 years to <6 years were defined as the group of 1–5 years, and so on. Notably, individuals with schizophrenia with durations of illness of 31 to 38 years were grouped together because of the relatively small sample size (*N* = 36). In addition, we compared the brain age gaps between each group of individuals with schizophrenia and age- and sex-matched healthy controls, which were selected randomly from the hold-out test dataset based on the sample size of each group of individuals with schizophrenia (Fig. 5c).

### Statistical analysis

For analysis of the clinicodemographic characteristics, the chi-square test and independent *t*-test were used for categorical and continuous variables, respectively.

Before group comparisons, we performed Tukey’s boxplot^72^ to identify and remove outlier data points for each group of individuals with schizophrenia. Values outside the interval [Q1-1.5*IQR and Q3 + 1.5*IQR] were considered outliers and removed. We performed the ANCOVA with the brain age gap as the dependent variable and diagnosis (i.e., each group of schizophrenia versus age- and sex-matched healthy controls from the hold-out test dataset) as the grouping variable. We included the chronological age, sex, MMSE score, and educational level as the covariates. The Bonferroni correction was used for five times comparisons, and the *p* value was set at 0.01. Partial eta squared was calculated as the effect size parameter in this study.

We also utilized Pearson’s correlation to examine the relationship of brain age gaps from three different trained models with clinical characteristics (i.e., the severity of symptoms and CPZ equivalent dosage), respectively.

## Supplementary information

Supplementary material

## Data Availability

The data presented here are not available due to a confidentiality agreement required by the institutional review board. All MATLAB scripts that were used in the analyses reported in this manuscript are available from the corresponding author upon request.
