# Supplementary material for: Predicting aging trajectories of decline in brain volume, cortical thickness and fractional anisotropy in schizophrenia
Source: Schizophrenia (Heidelb). 2023 Jan 3;9(1):1. doi: 10.1038/s41537-022-00325-w (PMC9810255; doi:10.1038/s41537-022-00325-w)
Supplement: Supplementary file 1 — Supplementary material [file 41537_2022_325_MOESM1_ESM.docx]

**Predicting Aging Trajectories of Decline in Brain Volume, Cortical Thickness and Fractional Anisotropy in Schizophrenia**

**– SUPPLEMENTARY MATERIAL –**

Jun-Ding Zhu, Shih-Jen Tsai, Ching-Po Lin, Yi-Ju Lee, Albert C. Yang*

**Supplementary Table 1. Demographic data for individuals with schizophrenia and age- and sex-matched healthy controls (hold-out test dataset) across different durations of illness**

| Duration of illness | Individuals with schizophrenia | | | | Healthy controls | | | |
| --- | --- | --- | --- | --- | --- | --- | --- | --- |
|  | Sample size | Sex,  Male (%) | Age | | Sample size | Sex,  Male (%) | Age | |
|  |  |  | Mean | SD |  |  | Mean | SD |
| 0 ~ 4 | 25 | 12 (48%) | 34.04 | 11.17 | 25 | 11 (44%) | 34.84 | 11.28 |
| 1 ~ 5 | 23 | 12 (52%) | 33.17 | 9.76 | 23 | 12 (52%) | 33.39 | 9.70 |
| 2 ~ 6 | 32 | 19 (59%) | 33.56 | 10.00 | 32 | 16 (50%) | 34.25 | 10.01 |
| 3 ~ 7 | 28 | 16 (57%) | 32.50 | 10.21 | 28 | 15 (54%) | 32.79 | 10.24 |
| 4 ~ 8 | 35 | 19 (54%) | 34.77 | 10.62 | 35 | 19 (54%) | 34.89 | 10.71 |
| 5 ~ 9 | 38 | 23 (61%) | 35.58 | 9.98 | 38 | 20 (53%) | 35.71 | 10.01 |
| 6 ~ 10 | 43 | 24 (56%) | 36.00 | 10.06 | 43 | 21 (49%) | 35.72 | 10.10 |
| 7 ~ 11 | 34 | 16 (47%) | 36.06 | 9.76 | 34 | 16 (47%) | 35.94 | 9.91 |
| 8 ~ 12 | 30 | 15 (50%) | 37.77 | 10.40 | 30 | 15 (50%) | 37.27 | 10.45 |
| 9 ~ 13 | 20 | 11 (55%) | 38.65 | 10.37 | 20 | 11 (55%) | 37.75 | 9.85 |
| 10 ~ 14 | 17 | 6 (35%) | 41.35 | 10.87 | 17 | 7 (41%) | 41.59 | 11.16 |
| 11 ~ 15 | 10 | 4 (40%) | 44.20 | 9.87 | 10 | 4 (40%) | 44.20 | 10.73 |
| 12 ~ 16 | 24 | 6 (25%) | 43.92 | 9.06 | 24 | 6 (25%) | 43.83 | 9.53 |
| 13 ~ 17 | 29 | 8 (28%) | 43.45 | 7.92 | 29 | 8 (28%) | 43.72 | 8.30 |
| 14 ~ 18 | 28 | 8 (29%) | 43.32 | 8.34 | 28 | 8 (29%) | 43.11 | 8.69 |
| 15 ~ 19 | 27 | 7 (26%) | 43.63 | 8.66 | 27 | 7 (26%) | 43.93 | 9.68 |
| 16 ~ 20 | 33 | 12 (36%) | 44.82 | 8.58 | 33 | 12 (36%) | 45.58 | 8.59 |
| 17 ~ 21 | 23 | 13 (57%) | 45.78 | 8.24 | 23 | 10 (43%) | 46.43 | 8.27 |
| 18 ~ 22 | 25 | 13 (52%) | 46.72 | 8.65 | 25 | 10 (40%) | 46.64 | 9.49 |
| 19 ~ 23 | 29 | 15 (52%) | 47.79 | 8.02 | 29 | 11 (40%) | 48.03 | 8.33 |
| 20 ~ 24 | 31 | 16 (52%) | 47.68 | 6.96 | 31 | 14 (45%) | 47.58 | 7.68 |
| 21 ~ 25 | 28 | 10 (36%) | 49.50 | 6.89 | 28 | 10 (36%) | 49.54 | 7.22 |
| 22 ~ 26 | 30 | 9 (30%) | 49.93 | 7.14 | 30 | 9 (30%) | 50.03 | 7.31 |
| 23 ~ 27 | 27 | 6 (22%) | 50.85 | 6.89 | 27 | 8 (30%) | 51.30 | 7.10 |
| 24 ~ 28 | 23 | 5 (22%) | 50.43 | 7.11 | 23 | 7 (30%) | 50.91 | 7.13 |
| 25 ~ 29 | 23 | 6 (26%) | 51.22 | 7.40 | 23 | 7 (30%) | 51.52 | 7.63 |
| 26 ~ 30 | 20 | 7 (35%) | 51.00 | 8.11 | 20 | 7 (35%) | 51.30 | 8.45 |
| 27 ~ 31 | 15 | 6 (40%) | 51.13 | 7.85 | 15 | 6 (40%) | 51.47 | 7.67 |
| 28 ~ 32 | 15 | 7 (47%) | 52.47 | 7.46 | 15 | 7 (47%) | 52.67 | 7.95 |
| 29 ~ 33 | 15 | 6 (40%) | 54.47 | 7.42 | 15 | 7 (47%) | 54.87 | 7.63 |
| 30 ~ 34 | 13 | 4 (31%) | 55.31 | 7.16 | 13 | 4 (31%) | 55.69 | 7.38 |
| 31 ~ 38 | 18 | 7 (39%) | 58.72 | 6.48 | 18 | 6 (33%) | 58.72 | 6.48 |

Abbreviations: SD = standard deviation

**Supplementary Table 2. Group differences in brain age gap between individuals with schizophrenia and healthy controls (hold-out test dataset) in the brain volume model.**

| Duration of illness | Individuals with schizophrenia | | Healthy controls | | ANCOVA | | |
| --- | --- | --- | --- | --- | --- | --- | --- |
|  | Mean | SD | Mean | SD | F | P value | Partial eta squared |
| 0 ~ 4 | 6.66 | 6.15 | -2.56 | 5.62 | 25.62 | **< 0.001** * | **0.37** |
| 1 ~ 5 | 6.77 | 4.85 | -1.86 | 5.63 | 23.34 | **< 0.001** * | **0.37** |
| 2 ~ 6 | 6.46 | 5.27 | -2.08 | 5.25 | 30.32 | **< 0.001** * | **0.34** |
| 3 ~ 7 | 5.27 | 6.39 | -1.41 | 6.26 | 12.83 | **0.001** * | **0.20** |
| 4 ~ 8 | 5.25 | 8.64 | -0.88 | 5.55 | 9.46 | **0.003** * | **0.13** |
| 5 ~ 9 | 4.75 | 8.30 | -0.78 | 5.42 | 11.26 | **0.001** * | **0.14** |
| 6 ~ 10 | 5.38 | 8.53 | -0.48 | 5.58 | 7.33 | **0.008** * | **0.08** |
| 7 ~ 11 | 5.76 | 8.69 | -1.73 | 5.36 | 9.38 | **0.003** * | **0.13** |
| 8 ~ 12 | 5.89 | 8.91 | -1.71 | 5.53 | 7.94 | **0.007** * | **0.13** |
| 9 ~ 13 | 5.16 | 8.02 | -1.94 | 5.53 | 7.38 | **0.010** * | **0.18** |
| 10 ~ 14 | 6.23 | 5.94 | -1.75 | 5.04 | 11.29 | **0.002** * | **0.29** |
| 11 ~ 15 | 2.83 | 4.26 | -5.96 | 5.14 | 10.95 | **0.005** * | **0.44** |
| 12 ~ 16 | 3.39 | 5.52 | -3.35 | 5.73 | 10.60 | **0.002** * | **0.20** |
| 13 ~ 17 | 4.05 | 7.83 | -3.02 | 6.28 | 10.94 | **0.002** * | **0.17** |
| 14 ~ 18 | 5.12 | 6.72 | -1.44 | 6.84 | 10.53 | **0.002** * | **0.17** |
| 15 ~ 19 | 5.50 | 7.50 | -1.57 | 6.78 | 8.06 | **0.007** * | **0.14** |
| 16 ~ 20 | 5.72 | 8.37 | -1.59 | 6.49 | 7.67 | **0.007** * | **0.11** |
| 17 ~ 21 | 5.74 | 9.62 | -4.04 | 5.94 | 10.33 | **0.003** * | **0.21** |
| 18 ~ 22 | 4.67 | 8.09 | -3.43 | 6.25 | 7.27 | **0.010** * | **0.14** |
| 19 ~ 23 | 4.76 | 7.92 | -2.95 | 7.06 | 8.65 | **0.005** * | **0.14** |
| 20 ~ 24 | 4.51 | 9.27 | -2.77 | 6.22 | 7.93 | **0.007** * | **0.12** |
| 21 ~ 25 | 5.82 | 10.00 | -1.38 | 7.59 | 7.28 | **0.009** * | **0.13** |
| 22 ~ 26 | 7.84 | 10.24 | -1.72 | 7.30 | 14.11 | **< 0.001** * | **0.21** |
| 23 ~ 27 | 8.61 | 10.44 | -1.28 | 7.13 | 14.76 | **< 0.001** * | **0.24** |
| 24 ~ 28 | 9.03 | 11.08 | -3.23 | 7.11 | 17.67 | **< 0.001** * | **0.31** |
| 25 ~ 29 | 9.70 | 8.92 | -2.60 | 7.07 | 17.53 | **< 0.001** * | **0.30** |
| 26 ~ 30 | 8.47 | 9.46 | -1.28 | 7.10 | 9.18 | **0.005** * | **0.21** |
| 27 ~ 31 | 8.39 | 6.82 | -2.55 | 6.58 | 13.43 | **0.001** * | **0.36** |
| 28 ~ 32 | 8.42 | 6.26 | -0.48 | 6.62 | 12.55 | **0.002** * | **0.34** |
| 29 ~ 33 | 9.97 | 4.82 | -2.50 | 6.72 | 15.24 | **0.001** * | **0.39** |
| 30 ~ 34 | 10.00 | 5.44 | -2.02 | 5.77 | 16.18 | **0.001** * | **0.45** |
| 31 ~ 38 | 9.04 | 6.57 | 1.67 | 6.23 | 11.91 | **0.002** * | **0.28** |
| Abbreviations: SD = standard deviation.  * represents significant difference after Bonferroni correction (p < 0.01). | | | | | | | |

**Supplementary Table 3. Group differences in brain age gap between individuals with schizophrenia and healthy controls (hold-out test dataset) in the cortical thickness model.**

| Duration of illness | Individuals with schizophrenia | | Healthy controls | | ANCOVA | | |
| --- | --- | --- | --- | --- | --- | --- | --- |
|  | Mean | SD | Mean | SD | F | P value | Partial eta squared |
| 0 ~ 4 | 8.55 | 9.01 | -5.68 | 6.82 | 31.93 | **< 0.001** * | **0.42** |
| 1 ~ 5 | 8.96 | 6.18 | -4.51 | 6.69 | 39.90 | **< 0.001** * | **0.50** |
| 2 ~ 6 | 8.95 | 6.30 | -4.36 | 7.18 | 38.07 | **< 0.001** * | **0.40** |
| 3 ~ 7 | 6.19 | 7.99 | -3.48 | 7.33 | 14.02 | **< 0.001** * | **0.22** |
| 4 ~ 8 | 5.84 | 8.26 | -2.03 | 7.58 | 10.32 | **0.002** * | **0.14** |
| 5 ~ 9 | 6.32 | 8.01 | -2.42 | 6.22 | 12.76 | **0.001** * | **0.15** |
| 6 ~ 10 | 6.42 | 7.67 | -2.67 | 7.29 | 19.17 | **< 0.001** * | **0.19** |
| 7 ~ 11 | 5.76 | 7.80 | -2.48 | 7.17 | 15.14 | **< 0.001** * | **0.20** |
| 8 ~ 12 | 5.84 | 8.32 | -1.12 | 7.47 | 8.68 | **0.005** * | **0.14** |
| 9 ~ 13 | 5.50 | 4.28 | -1.48 | 7.64 | 10.84 | **0.002** * | **0.24** |
| 10 ~ 14 | 6.49 | 7.87 | -3.43 | 4.65 | 19.15 | **< 0.001** * | **0.41** |
| 11 ~ 15 | 7.97 | 9.48 | -3.86 | 7.00 | 13.43 | **0.003** * | **0.49** |
| 12 ~ 16 | 6.97 | 8.61 | -2.43 | 7.13 | 14.01 | **0.001** * | **0.25** |
| 13 ~ 17 | 6.56 | 8.26 | -3.73 | 6.99 | 21.92 | **< 0.001** * | **0.30** |
| 14 ~ 18 | 6.67 | 8.12 | -2.29 | 7.42 | 14.24 | **< 0.001** * | **0.22** |
| 15 ~ 19 | 7.19 | 6.94 | -3.25 | 7.77 | 21.90 | **< 0.001** * | **0.31** |
| 16 ~ 20 | 7.61 | 7.43 | -4.42 | 7.46 | 26.92 | **< 0.001** * | **0.31** |
| 17 ~ 21 | 7.77 | 7.51 | -3.54 | 8.10 | 18.51 | **< 0.001** * | **0.32** |
| 18 ~ 22 | 8.71 | 7.03 | -4.75 | 7.57 | 32.13 | **< 0.001** * | **0.42** |
| 19 ~ 23 | 9.24 | 6.63 | -2.74 | 6.86 | 26.75 | **< 0.001** * | **0.34** |
| 20 ~ 24 | 9.01 | 7.55 | -1.32 | 7.50 | 18.19 | **< 0.001** * | **0.25** |
| 21 ~ 25 | 8.42 | 7.75 | -2.82 | 7.30 | 20.39 | **< 0.001** * | **0.29** |
| 22 ~ 26 | 9.11 | 7.97 | -2.76 | 6.68 | 29.84 | **< 0.001** * | **0.36** |
| 23 ~ 27 | 7.91 | 7.71 | -3.21 | 6.97 | 23.73 | **< 0.001** * | **0.33** |
| 24 ~ 28 | 6.67 | 8.23 | -1.10 | 6.61 | 11.69 | **0.001** * | **0.23** |
| 25 ~ 29 | 5.88 | 7.69 | -1.99 | 7.68 | 10.18 | **0.003** * | **0.20** |
| 26 ~ 30 | 4.97 | 8.28 | -3.40 | 7.41 | 8.43 | **0.006** * | **0.20** |
| 27 ~ 31 | 4.11 | 7.68 | -4.96 | 5.87 | 8.29 | **0.008** * | **0.26** |
| 28 ~ 32 | 5.25 | 8.05 | -4.19 | 8.45 | 8.10 | **0.009** * | **0.25** |
| 29 ~ 33 | 6.71 | 7.95 | -3.26 | 7.88 | 14.00 | **0.001** * | **0.37** |
| 30 ~ 34 | 6.98 | 7.05 | -4.14 | 8.48 | 11.09 | **0.003** * | **0.36** |
| 31 ~ 38 | 8.57 | 7.52 | 0.77 | 9.37 | 7.87 | **0.009** * | **0.21** |
| Abbreviations: SD = standard deviation.  * represents significant difference after Bonferroni correction (p < 0.01). | | | | | | | |

**Supplementary Table 4. Group differences in brain age gap between individuals with schizophrenia and healthy controls (hold-out test dataset) in the fractional anisotropy model.**

| Duration of illness | Individuals with schizophrenia | | Healthy controls | | ANCOVA | | |
| --- | --- | --- | --- | --- | --- | --- | --- |
|  | Mean | SD | Mean | SD | F | P value | Partial eta squared |
| 0 ~ 4 | -1.91 | 9.38 | -1.45 | 6.74 | 0.01 | **0.919** | **< 0.01** |
| 1 ~ 5 | -0.64 | 8.11 | -1.05 | 7.34 | 0.00 | **0.975** | **< 0.01** |
| 2 ~ 6 | -0.55 | 8.01 | -1.30 | 7.68 | 0.01 | **0.928** | **< 0.01** |
| 3 ~ 7 | -0.28 | 8.14 | 0.40 | 6.71 | 0.72 | **0.400** | **0.01** |
| 4 ~ 8 | 0.92 | 8.83 | 0.54 | 7.17 | 0.05 | **0.821** | **< 0.01** |
| 5 ~ 9 | 0.57 | 8.70 | -0.88 | 7.07 | 0.15 | **0.700** | **< 0.01** |
| 6 ~ 10 | 0.61 | 8.58 | -1.26 | 7.45 | 0.41 | **0.523** | **0.01** |
| 7 ~ 11 | 1.00 | 8.73 | -1.16 | 8.22 | 1.07 | **0.306** | **0.02** |
| 8 ~ 12 | 1.52 | 8.81 | -0.20 | 7.17 | 0.41 | **0.525** | **0.01** |
| 9 ~ 13 | -1.58 | 6.18 | -2.45 | 8.37 | 1.01 | **0.323** | **0.03** |
| 10 ~ 14 | -2.93 | 5.17 | -0.50 | 9.01 | 0.24 | **0.629** | **0.01** |
| 11 ~ 15 | -2.96 | 6.67 | -1.13 | 6.47 | 0.11 | **0.741** | **0.01** |
| 12 ~ 16 | -1.69 | 7.26 | -2.37 | 7.20 | 0.23 | **0.633** | **0.01** |
| 13 ~ 17 | -1.77 | 6.39 | -2.68 | 6.64 | 0.67 | **0.416** | **0.01** |
| 14 ~ 18 | -1.41 | 7.76 | -1.63 | 8.18 | 0.16 | **0.692** | **< 0.01** |
| 15 ~ 19 | -1.51 | 8.13 | -0.98 | 7.87 | 0.00 | **0.957** | **< 0.01** |
| 16 ~ 20 | 0.86 | 8.44 | -0.23 | 7.38 | 0.29 | **0.590** | **< 0.01** |
| 17 ~ 21 | 3.69 | 7.67 | 1.04 | 6.93 | 1.99 | **0.166** | **0.05** |
| 18 ~ 22 | 2.27 | 8.87 | -0.02 | 8.24 | 0.02 | **0.882** | **< 0.01** |
| 19 ~ 23 | 1.57 | 8.93 | -0.87 | 8.29 | 1.84 | **0.181** | **0.03** |
| 20 ~ 24 | 2.28 | 8.92 | -0.15 | 6.43 | 3.76 | **0.058** | **0.06** |
| 21 ~ 25 | 1.03 | 8.48 | -0.50 | 7.92 | 1.74 | **0.193** | **0.03** |
| 22 ~ 26 | 1.67 | 9.95 | 0.57 | 7.14 | 0.93 | **0.340** | **0.02** |
| 23 ~ 27 | 1.91 | 10.66 | -0.47 | 7.41 | 1.80 | **0.186** | **0.04** |
| 24 ~ 28 | 3.54 | 10.05 | -0.85 | 8.53 | 2.20 | **0.145** | **0.05** |
| 25 ~ 29 | 2.80 | 10.48 | -1.86 | 7.24 | 1.39 | **0.245** | **0.03** |
| 26 ~ 30 | 3.28 | 10.57 | 1.15 | 8.09 | 0.34 | **0.565** | **0.01** |
| 27 ~ 31 | 2.21 | 8.97 | 1.01 | 7.48 | 0.06 | **0.811** | **< 0.01** |
| 28 ~ 32 | 0.98 | 7.18 | 2.80 | 5.49 | 3.23 | **0.085** | **0.12** |
| 29 ~ 33 | 1.56 | 7.55 | 1.47 | 7.54 | 0.00 | **0.951** | **< 0.01** |
| 30 ~ 34 | 1.36 | 5.60 | 3.28 | 7.58 | 0.15 | **0.705** | **0.01** |
| 31 ~ 38 | 3.88 | 6.31 | 3.35 | 6.35 | 0.26 | **0.615** | **0.01** |
| Abbreviations: SD = standard deviation.  * represents significant difference after Bonferroni correction (p < 0.01). | | | | | | | |

**Supplementary Table 5. Clinicodemographic characteristics of the training dataset and the hold-out test dataset.**

| Characteristics | Training dataset  (n = 230) | Hold-out test dataset  (n = 100) | Statistic  (t or $\chi$^2^) | p value |
| --- | --- | --- | --- | --- |
| Sex  Male, n (%)  Female, n (%) | 93 (40.4%)  137 (59.6%) | 36 (36.0%)  64 (64.0%) | 0.41 | 0.53 ^b^ |
| Age, year  (Range) | 43.12 ± 15.72  (20–84) | 44.34 ± 14.91  (21–74) | –0.66 | 0.51 ^a^ |
| 20–29  Male/female, n  Total, n (%) | 32/37  69 (30.0%) | 10/13  23 (23.0%) | < 0.01 | > 0.99 ^b^ |
| 30–39  Male/female, n  Total, n (%) | 15/21  36 (15.7%) | 6/8  14 (14.0%) | < 0.01 | > 0.99 ^b^ |
| 40–49  Male/female, n  Total, n (%) | 14/21  35 (15.2%) | 7/13  20 (20.0%) | 0.01 | 0.94 ^b^ |
| 50–59  Male/female, n  Total, n (%) | 15/28  43 (18.7%) | 11/14  25 (25.0%) | 0.24 | 0.63 ^b^ |
| 60–69  Male/female, n  Total, n (%) | 15/27  42 (18.3%) | 2/14  16 (16.0%) | 0.16 | 0.11 ^b^ |
| 70–79  Male/female, n  Total, n (%) | 0/1  1 (0.4%) | 0/2  2 (2.0%) | - | - |
| 80–89  Male/female, n  Total, n (%) | 2/2  4 (1.7%) | 0/0  0 (0%) | - | - |
| Education level, year | 15.82 ± 3.81 | 15.93 ± 3.95 | –0.22 | 0.82 ^a^ |
| MMSE | 29.00 ± 0.98 | 28.88 ± 1.09 | 0.95 | 0.34 ^a^ |

Data are mean ± SD or n (%) unless specified otherwise. Abbreviations: MMSE, Mini-Mental State Examination.

^a^ Independent *t* test, significance level = 0.05.

^b^ Chi-square test, significance level = 0.05.


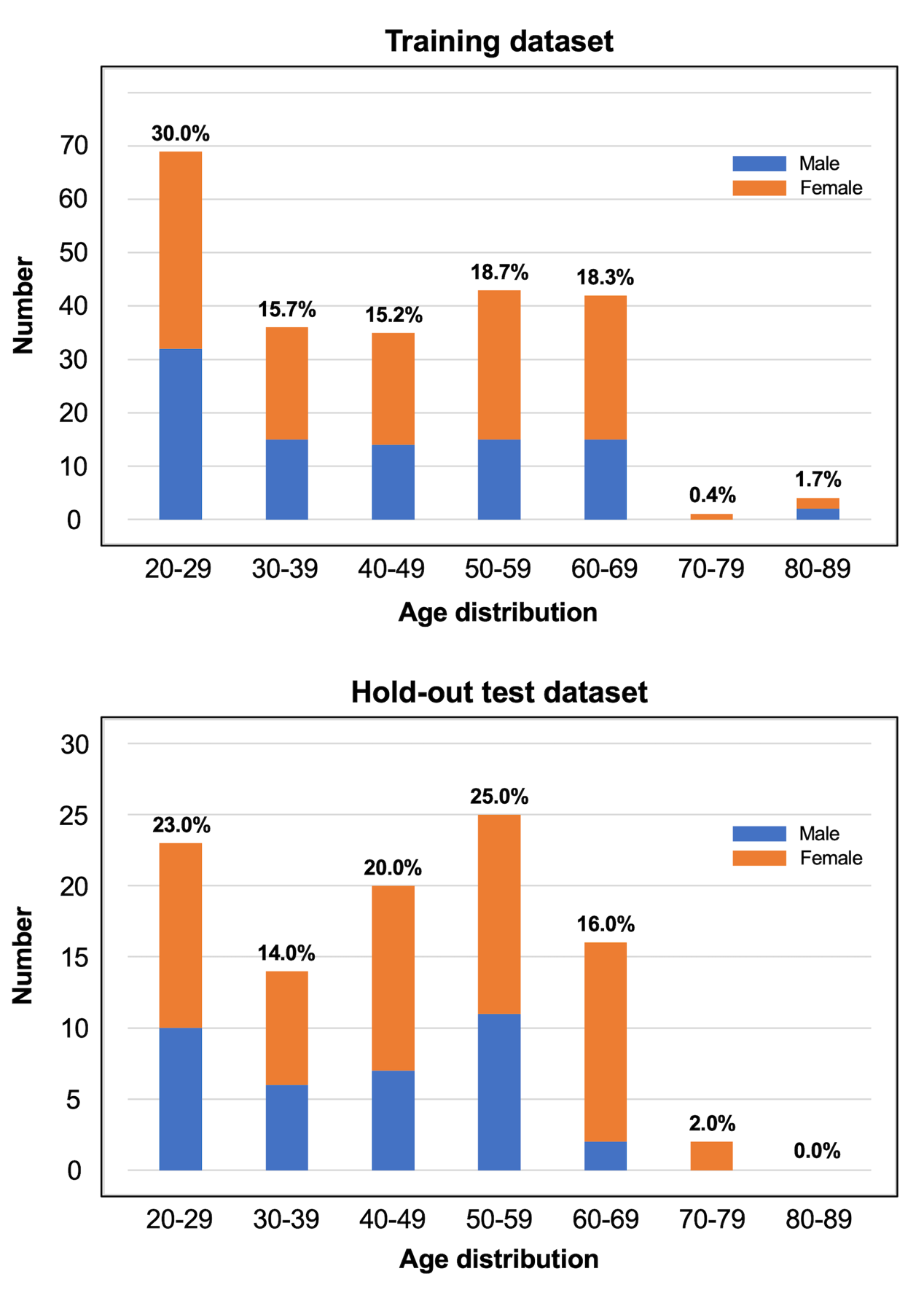


**Supplementary Figure 1. Age distributions of training and hold-out test datasets.**
